# Editorial for the Special Issue on Advances in Optoelectronic Devices, 2nd Edition

**DOI:** 10.3390/mi15080995

**Published:** 2024-07-31

**Authors:** Feng Li, Zichuan Yi, Jiashuai Wang, Mouhua Jiang

**Affiliations:** 1 School of Electronic Information, University of Electronic Science and Technology of China, Zhongshan Institute, Zhongshan 528402, China; 202322310333@std.uestc.edu.cn (F.L.); 202221021120@std.uestc.edu.cn (J.W.); 2022024132@m.scnu.edu.cn (M.J.); 2 School of Integrated Circuit Science and Engineering (Exemplary School of Microelectronics), University of Electronic Science and Technology of China, Chengdu 611731, China

Optoelectronic devices have improved people’s quality of life and have received widespread attention for a long time. Since the beginning of the 21st century, the application fields of optoelectronic devices have been continuously expanding, and their market share has been rising. However, there is still room for improvement in the performance of many optoelectronic devices. Therefore, with the development of society, people have higher and higher requirements for the performance of optoelectronic devices. Many researchers are engaged in the field of optoelectronic devices and attempt to contribute to this field. This Special Issue includes nine papers, covering five branches of optoelectronic devices: optical displays, lasers, solar cells, waveguides, and optoelectronic sensors. The latest research progress is discussed from the perspectives of algorithms, materials, and structures.

Electronic paper, an innovative display technology, is dedicated to simulating the reading experience of traditional paper. Its advantages include low power consumption and high readability. Electronic paper not only provides reading comfort similar to paper, but also has a broad perspective and good durability. These advantages make it an ideal display screen choice for E-book readers, public information display devices, smartwatches, and wearable devices. The core component of electronic paper is the electrophoretic display. It is composed of microcapsules containing positive and negative charged pigment particles, and the particles are rearranged to display images and text through the action of an electric field [1]. Electrowetting display is the most likely alternative to electrophoretic display for electronic paper. It controls the display content by changing the shape of the liquid in pixels. This technology has the characteristics of low power consumption, high contrast, and a wide viewing angle; it makes up for the slow response speed of electrophoretic displays [2]. However, both electrophoretic displays and electrowetting displays currently have performance limitations. Electrophoretic displays have issues such as slow refresh rates and limited color display capabilities. The focus of color display is the selection of electrophoretic particles. In this Special Issue, Zhang et al. successfully used ionic liquid to chemically modify the surface of 181 yellow pigment particles through sol-gel technology and a surface grafting process, so that SiO_2_ microspheres successfully formed a coating on the surface of PY181 particles, and constructed charged yellow pigment particles with a core-shell structure [3]. The results indicated that the modified yellow pigment particles exhibit significant electrophoretic responsiveness, which effectively enhanced the color display capability of electrophoretic electronic paper. Furthermore, this study provided an innovative solution strategy for improving the electrophoretic behavior of colored pigment particles which were difficult to directly bind with ionic liquids. This technology is expected to be widely applied in the field of the surface modification of pigment particles. There are still many issues regarding the electrowetting display, such as oil reflux, oil splitting, and charge capture, which seriously restrict its display performance. After analyzing the driving principle of electrowetting displays, this Special Issue proposed a more efficient driving waveform, which was divided into two stages [4]. The first stage was an initial fast driving stage. At this stage, it quickly adjusted the display content by using exponential function waveforms. The role of the second stage was to stabilize the display image. To enhance the stability of the display effect, it utilized AC pulse signals to release accumulated charges on the interface between the hydrophobic insulation layer and ink contact. The results indicated that the driving waveform could suppress oil reflux and brightness oscillation at different grayscale levels. The above research provided a direction for improving the performance of electronic paper displays and promoted the development of optoelectronic technology in the field of visual displays.

With the increasing application of laser diode pumped solid-state lasers (DPSSLs), the key structure for heat dissipation, side pumped amplifiers, has received widespread attention [5]. Moreover, in the field of lasers, butterfly packaged lasers are becoming increasingly popular due to their wide range of applications [6]. Coupling efficiency, as a key parameter of butterfly packaged lasers, affects laser output and communication performance directly. The key to coupling efficiency lies in the number of particles used in the population optimization strategy [7]. In terms of DPSSLs, Nie et al. used simulation software to numerically simulate the structure of the laser diode array side pump amplifier in this Special Issue [8]. With this as a premise, they created a fluid structure coupling model. Subsequently, they studied and explored the relationship between the relevant parameters of the model and its performance. The experimental results indicated that increasing the coolant flow rate could reduce the maximum temperature and equivalent stress in the laser system. At low flow rates, the thickness and dosage of the cooling layer had limited effects on improving the heat dissipation efficiency. At high flow rates, changes in the thickness of the cooling layer had little effect on temperature and force. These results provided optimization guidance for the design of laser amplifiers and DPSSLs systems. In the aspect of butterfly packaged lasers, this Special Issue developed a chaotic adaptive seeker optimization algorithm (CASOA) [9]. To improve coupling efficiency, this algorithm adopted a population optimization strategy with fewer particles. The results indicated that the proposed optimization method had high efficiency and precision in optimizing situations with small population sizes. This method could quickly locate with only a few iterations while maintaining 100% search accuracy. Traditional optimization strategies reduced the number of iterations and improved coupling efficiency by increasing the population appropriately, but they may increase motion errors and displacement. The smaller population strategies of CASOA reduced these errors, thereby optimizing the coupling alignment of the laser diode and ultimately improving the overall coupling efficiency.

In order to break free from the constraints of fossil fuels, photovoltaic power generation has developed and become an important component of photovoltaic applications [10]. As the main body of a photovoltaic power generation system, the importance of solar cells is self-evident. People have never stopped searching for better solar cell materials, so organic solar cells have been widely studied due to their excellent performance [11]. The semi- transparent organic solar cell (ST-OSC), as its representative type, is also the expectation of everyone [12]. With the development of photovoltaic energy, many new problems have also emerged. The continuous expansion of photovoltaic power generation networks has led to an increase in photovoltaic system failures. So, this Special Issue raised a method for locating the location and type of faults in photovoltaic networks quickly [13]. This method mainly involved three processes: detecting abnormal signals, separating errors, and detecting fault types. It utilized a fuzzy logic control structure to handle uncertain problems, and intelligent algorithms were used to optimize parameters, thereby improving the accuracy of the detection system and enhancing detection speed. The results showed that this method could improve the localization speed and accuracy of photovoltaic network faults effectively. In terms of solar cell materials, organic solar cells using solution treatment processes have achieved significant breakthroughs in power conversion efficiency (PCE). One of them, semi-transparent organic solar cells, has shown great potential in multiple fields such as agricultural greenhouses and wearable devices. In this Special Issue, researchers have developed high-performance ST-OSCs through continuous optimization of transparent electrodes, as well as continuous innovation in materials and structures [14]. The PCE and average visible light transmittance of these batteries have been significantly improved. And this new type of semi-transparent organic solar cell is expected to drive a crucial reform in photovoltaic power generation.

Due to the increasing demand for data services, people have higher requirements for the distance and speed of data transmission. Optical links have entered people’s lives due to their excellent communication performance [15]. Waveguides as carriers of optical links are also receiving increasing attention, but the doping of waveguides is also influenced by silicon modulators [16]. In response to the problem of low modulation efficiency in traditional modulators, this Special Issue presents an improved-performance X-ray silicon modulator with a three-dimensional profile [17]. This modulator utilized two reverse tilted junctions combined with an effective three-dimensional Monte Carlo method to generate a junction. The results indicated that three-dimensional doping structures could bring many advantages to silicon modulators. This structure could not only achieve high modulation efficiency and low loss three-dimensional junction design, but also effectively reduce the resistance and capacitance of the modulator. These improvements have enabled 3D doping structures to play an important role in improving the overall performance of silicon-based modulators, thereby driving the development of modulator technology to a higher level. The waveguide prepared using this technology mainly consisted of two parts in structure: metal-based cylindrical hybrid waveguide and silver nanowire. Moreover, this Special Issue had specially designed two coupling regions, significantly increasing coupling strength [18]. The final results indicated that the waveguide has good transmission efficiency and quality. In addition, the cross-sectional area of the waveguide was very small, so it could perform precise optical operations on extremely small scales, which greatly contributes to the improvement in device integration.

Finally, in terms of photoelectric sensors, this Special Issue studied and improved surface-enhanced Raman spectroscopy (SERS) technology, which has the advantage of high sensitivity [19]. This technology combined patterned sapphire substrates (PSSs) with micro-cone arrays and conducted self-assembly and surface deposition reactions on polystyrene (PS) nanospheres. Then, a nanoscale three-dimensional silver bowl array was formed. By optimizing the reaction time, the SERS performance and structural characteristics were improved. The results indicated that the patterned PSS substrate had significant advantages in capturing light compared to traditional planar substrates.

Currently, people’s lives are inseparable from optoelectronic device; everyone has high expectations for the performance of optoelectronic devices. Based on nine papers related to optoelectronic devices, this Special Issue discusses the latest development of optoelectronic devices. It also provides optimization solutions of optoelectronic devices in terms of material optimization, structure innovation, algorithm optimization, and other aspects, hopefully providing help for future scientific research and industrial development.

## Data Availability

Some data are available from the authors upon request.
